# Integrating Omics Data in Genome-Scale Metabolic Modeling: A Methodological Perspective for Precision Medicine

**DOI:** 10.3390/metabo13070855

**Published:** 2023-07-18

**Authors:** Partho Sen, Matej Orešič

**Affiliations:** 1 Turku Bioscience Centre, University of Turku and Åbo Akademi University, FI-20520 Turku, Finland; 2 School of Medical Sciences, Faculty of Medicine and Health, Örebro University, 702 81 Örebro, Sweden

**Keywords:** constraint-based modeling, host microbiome, human metabolism, human metabolic networks, metabolic reconstructions, metabolic modeling, multi-omics

## Abstract

Recent advancements in omics technologies have generated a wealth of biological data. Integrating these data within mathematical models is essential to fully leverage their potential. Genome-scale metabolic models (GEMs) provide a robust framework for studying complex biological systems. GEMs have significantly contributed to our understanding of human metabolism, including the intrinsic relationship between the gut microbiome and the host metabolism. In this review, we highlight the contributions of GEMs and discuss the critical challenges that must be overcome to ensure their reproducibility and enhance their prediction accuracy, particularly in the context of precision medicine. We also explore the role of machine learning in addressing these challenges within GEMs. The integration of omics data with GEMs has the potential to lead to new insights, and to advance our understanding of molecular mechanisms in human health and disease.

## 1. Introduction

The integration of omics data such as genomics, transcriptomics, proteomics, and metabolomics has revolutionized our understanding of biological systems by providing a holistic view of the complex molecular processes associated with human health [1]. Genome-scale metabolic modeling (GSMM) is a constraint-based mathematical modeling technique that has been instrumental in the analysis of omics data [2,3]. Genome-scale metabolic models (GEMs) provide a robust framework that enables the integration of multiple omics datasets [4,5]. By harnessing the power of GEMs, researchers can delve into the complexities of biological pathways, enabling a comprehensive understanding of cellular metabolism and its underlying mechanisms [2,6,7,8,9]. Furthermore, incorporating biochemical and genetic information into GEMs has enabled researchers to understand the interplay between genes, proteins, and metabolites involved in cellular metabolism. This integrative approach effectively bridges the gap between genotypes and phenotypes [4,5,10].

GEMs serve as a valuable tool for predicting metabolic capabilities and identifying key regulatory nodes in biological systems, representing a paradigm shift in omics data analysis. GSMM has been used to study gut ecosystems, exploring the metabolic interactions between the host and the microbial communities in the gut [11,12,13,14]. Notably, the development of GEMs for catalogued human gut microbes [15,16] by uncovering their metabolic functions has been a major recent achievement. 

In this review, we explore how GSMM has contributed to our understanding of human metabolism. Specifically, we focus on two important approaches: (a) tissue-specific modeling and (b) host-microbiome modeling. We address key challenges associated with model reconstruction and validation to improve the reproducibility and reliability of GEM-based predictions. In addition, we emphasize the wide-ranging scope and diverse applications of GEMs in the field of precision medicine.

## 2. Exploring Human Metabolism: The Evolution of Genome-Scale Metabolic Models

Throughout the past three decades, GEMs have undergone continuous evolution, deepening our understanding of metabolic processes and their implications in various biological and clinical studies [9,17]. GEMs have been widely used in metabolic engineering, where they have demonstrated their ability to predict cellular growth under different nutrient conditions [18,19]. Furthermore, they have been applied to design studies, investigating the essentiality of reactions/genes [20,21] and the relevance of metabolic pathways [22] and modeling phenotypes by manipulating these pathway(s) [23]. 

Over the past 15 years, there has been a focused and continuous effort by researchers to develop and enhance GEMs for human metabolism. Recon 1 was one of the first generic reconstructions of human metabolism, which aimed to integrate and analyze diverse biological datasets, providing a foundation for studying human metabolic pathways [24]. EHMN (Edinburgh Human Metabolic Network) was developed to capture the intricacies of human metabolism and facilitate comprehensive analyses of metabolic functions. EHMN served as a valuable resource for exploring metabolic interactions and pathways in humans [25]. Following that, Recon 2 was built upon Recon 1, leading to an expanded coverage of human metabolic pathways and thereby offering an enhanced understanding of metabolic processes in health and disease [26,27]. HMR (Human Metabolic Reaction) encompasses a comprehensive collection of metabolic reactions, enzymes, and associated genes. It serves as a valuable resource for studying metabolic networks [6,28]. Recently, Recon 3D, a three-dimensional reconstruction of human metabolism, was developed, which integrates spatial information to better represent the complexity of metabolic reactions within cellular compartments. Recon 3D provides a detailed and context-specific view of human metabolism, enabling deeper insights into cellular function than previously possible [3]. HMR and Recon reconstructions have been extensively utilized to study various diseases, such as type 2 diabetes (T2D), non-alcoholic fatty liver disease (NAFLD), cancer, and immunometabolism [6,29,30,31,32]. In addition, Robinson et al. developed Human1 [33], a unified Human-GEM, and a web portal, Metabolic Atlas (https://metabolicatlas.org/ (accessed on 23 July 2015)), by integrating and curating the Recon and HMR model’s lineages. The entire development process followed a systematic approach. Moreover, Human1 and Metabolic Atlas were demonstrated to be valuable tools in identifying metabolic vulnerabilities, for instance, in acute myeloid leukemia; predicting essential genes for specific metabolic functions; and estimating metabolic fluxes and growth rates. These advancements enhance the capacity of GEMs to model metabolic pathways associated with health and disease.

Taken together, genome-scale metabolic reconstructions have contributed to our understanding of metabolism, providing a foundation for studying metabolic pathways, predicting regulations, and exploring the impact of genetic variations and environmental factors on human health.

## 3. Integrating Omics Data into Genome-Scale Metabolic Models: Overcoming Challenges and Shaping Perspectives

Omics data have been extensively used to deepen our understanding of various biological systems, including cells, tissues, and organs. By incorporating individual- or condition-specific omics datasets, researchers can develop models that accurately represent the metabolic characteristics of an individual at a particular condition. This approach allows for a more precise investigation of metabolic pathways and therefore provides a foundation for precision medicine and the selection of optimal treatment strategies [34,35]. Furthermore, overlaying omics data with the metabolic networks has enabled researchers to infer metabolic regulations and identify patterns associated with metabolic states [7,8,28,36]. 

The integration of omics data poses several challenges, and addressing these challenges requires continuous advancements in data acquisition, standardization, computational methodologies, and model validation techniques to enhance the accuracy and applicability of integrated models in various biological contexts.

A vast amount of data makes their integration and harmonization a daunting task. Diverse data types, formats, and measurement scales require meticulous effort with respect to data integration and standardization. The heterogeneity of data sources, i.e., datasets generated from different studies, experiments, or platforms, introduces variations in the data. Managing these technical variations is crucial to ensure the consistency of the results. Moreover, quality control measures such as outlier removal, artifact correction, and noise filtering are undertaken to improve data quality [1,37,38,39]. Dealing with the missing values is a critical aspect of omics data that can undermine the accuracy and reliability of the outcomes, if not properly adjusted for. To mitigate this issue, imputation methods are commonly employed to estimate missing values and minimize the impact of data sparsity [40]. Lastly, normalization plays an important role in standardizing the scale and range of omics data across different samples or conditions [41,42]. The normalization of omics data involves the utilization of various tools, with each suited to specific omics data types and analytical requirements. Central tendency-based normalization, such as mean and median, is a simple yet effective method employed for normalizing proteomics and metabolomics data. It rescales the intensity values of individual samples to align with the mean or median intensity across all samples. Quantile normalization [43] is a widely used technique for normalizing gene expression data derived from microarrays. By aligning the empirical distributions of expression values across samples, it ensures comparability and robustness. For high-throughput genomic data, including microarrays or DNA methylation arrays, *ComBat* [44] is a specialized tool designed to address batch effects. By employing an empirical Bayes framework, *ComBat* effectively adjusts for batch-related variations, leading to more reliable results. *ComBat-seq* [45] is an advanced method that utilizes a negative binomial regression model to address batch effects in RNA-seq studies. *RUVSeq* (remove unwanted variation in RNA-seq) is a valuable tool dedicated to eliminating the unwanted sources of variations in RNA-seq data, such as batch effects or confounding factors [46]. Leveraging a factor analysis-based approach, *RUVSeq* estimates and removes these sources of variation, enhancing the accuracy of downstream analyses. *Limma* and *Limma-Voom* are versatile tools that are extensively used for normalizing gene expression microarray data and RNA-seq data, respectively. These tools utilize linear modeling and empirical Bayes methods to effectively handle technical variations and batch effects, ensuring robust and accurate analysis. *DESeq2* [47] is a widely adopted tool that is specifically designed for normalizing RNA-seq data. By utilizing a negative binomial distribution model, *DESeq2* effectively accounts for sequencing depth and sample-specific biases, enabling reliable differential expression analysis. In addition, *edgeR* [48] is another popular tool employed for RNA-seq data normalization. It utilizes a robust empirical Bayes approach to estimate normalization factors, considering library size and gene-specific biases. Next-generation sequencing (NGS) data can be normalized using multiple methods such as TMM (trimmed mean of M values), RPKM (reads per kilobase per million mapped reads), and CPM (counts per million), utilizing tools such as *DESeq2*, *edgeR*, and *limma*. Furthermore, *NOMIS* [49], a normalization method that is specifically developed for metabolomics data, employs the optimal selection of multiple internal standards to ensure accurate and reliable normalization. A thorough description of normalization methods applied to omics data is discussed elsewhere [41,50].

Furthermore, omics data may not capture the entire metabolic network or pathway, leading to incomplete coverage of the GEMs. Missing or unmeasured components can limit the model’s accuracy and predictive capabilities, particularly in scenarios where these components play critical roles. 

Integrating omics data into GSMMs involves complex mathematical and computational algorithms. The process requires sophisticated tools and expertise to handle large datasets, perform data preprocessing, and apply appropriate integration methods, which can be computationally demanding.

Several standalone software suites, such as *COBRA* (constraint-based reconstruction and analysis) [51,52,53], *Microbiome Modeling Toolbox* [54], *FastMM* (a toolbox for personalized constraint-based metabolic modeling) [55], *rBioNet* [56], and *RAVEN* (reconstruction, analysis, and visualization of metabolic networks) [57,58], offer comprehensive functionalities for metabolic reconstructions, modeling, and the integration of omics data. A comprehensive list of the resources used for human metabolic pathway reconstructions is given in (Table 1).

## 4. Modeling Tissue-Specific Interactions: Integrating Omics Data for Contextualization

Tissue-specific GEMs have emerged as powerful tools for investigating the complex metabolic processes that occur within specific cell or tissue types [6,8,80]. These models are reconstructed by integrating various types of omics data, including gene, transcript, protein, and metabolite data, allowing for a more nuanced and context-specific representation of the metabolic networks within a specific cell type. To leverage expression data, several algorithms have been developed. *GIMME* (gene inactivity moderated by metabolism and expression) utilizes gene expression or transcriptomics data to predict active reactions in a GEM [81,82], aiming to identify a consistent set of metabolic reactions that are active under the given conditions. This approach provides insights into the metabolic activity of specific cell-types. Another notable algorithm, *iMAT* (integrative metabolic analysis tool), integrates gene expression data to identify active metabolic reactions [83]. By combining transcriptomic data with GEMs, it formulates an optimization problem to determine the set of reactions that is likely to be active, enabling the identification of condition-specific metabolic activity and offering a valuable framework for understanding tissue-specific metabolism. The *MADE* (metabolic adjustment by differential expression) algorithm leverages gene expression data to predict metabolic adaptations in response to different conditions [84]. By considering changes in gene expression levels and utilizing a network-based approach, *MADE* identifies reactions that are differentially regulated between conditions, shedding light on how the metabolic network adjusts its activity in response to cellular or environmental changes. The *E-flux* algorithm incorporates gene expression data to estimate the metabolic flux distribution within a tissue-specific GEM [85]. By combining gene expression profiles with flux balance analysis (FBA), *E-flux* estimates the distribution of metabolic fluxes and predicts the activity of specific reactions, offering a valuable tool for understanding the flow of metabolites through metabolic pathways in specific tissues. The *tINIT* (transcriptional integration of tissue-specific GEMs) algorithm integrates gene expression data with a GEM to predict active metabolic reactions [80,86]. By considering gene expression levels and regulatory interactions, *tINIT* estimates the transcriptional activity of metabolic genes and identifies active reactions within the metabolic network, providing a deeper understanding of tissue-specific metabolic activity by incorporating transcriptional regulation. The *METRADE* (metabolic and transcriptomics adaptation estimator) framework offers a comprehensive approach for integrating gene and protein expression data, allowing the combination of both types of omics data and enabling a more holistic understanding of biological processes [87]. Similarly, the *IOMA* (integrative omics-metabolic analysis) platform provides an opportunity to integrate proteomic and metabolomic data [88]. Other algorithms include *FASTCORE* [89], *FASTCORMICS* [90], *mCADRE* [91], *PRIME* [92], *RegrEX* [93], and *CORDA* [94], and a detail description of these methods, including their implementation, characteristics, and applications, is reviewed elsewhere [4,95]. 

Automated methodologies have been utilized to develop tissue- and cell-specific models by combining human metabolic reconstructions, primarily from HMR and Recon lineages, along with omics data [80,91]. Semi-automated techniques for model reconstructions are included in software suites such as *COBRA* [51,52,53] and *RAVEN* [57,58], which offer advantages in terms of speed and throughput, enabling the construction of large-scale models within a reasonable time frame. However, it is important to note that the speed and throughput may vary depending on the specific methodologies employed and the complexity of the model being constructed. While semi-automated methods provide valuable efficiencies, they may also have some limitations. These include a reliance on manual curation and expert knowledge, which can introduce subjectivity and potential biases into the model development process [2,96]. Additionally, the need for manual intervention and decision making during the model’s construction phase may introduce human error. To facilitate the automated reconstruction of GEMs, web-servers like *KBase* [97] and *ModelSEED* [61] have been designed to incorporate large datasets. However, the transition from semi-automated to fully automated approaches face certain barriers. One significant barrier is the requirement for high-quality and comprehensive data, as well as accurate and reliable algorithms, to automate the model generation process effectively. Furthermore, the dynamic and context-specific nature of biological systems poses challenges in capturing the full complexity of metabolic networks in a fully automated manner. Overcoming these barriers requires refining computational techniques to handle large-scale data integration and model construction, ensuring the accuracy and reliability of automated approaches. 

Notable examples of tissue-specific models include liver GEMs that are specifically tailored to mimic the metabolism of the human liver [6,8,98,99]. In a recent study, the GEMs of the human liver have been developed to examine patients with NAFLD at different stages of fibrosis [8]. The study revealed metabolic signatures related to vitamins (A and E), glycosphingolipids, and complex glycosaminoglycans in advanced fibrosis. Furthermore, the models identified metabolic patterns that are associated with three gene variants (*PNPLA3, TM6SF2,* and *HSD17B13*) linked to NAFLD, providing insights into metabolic dysregulation in the liver and its contribution towards NAFLD progression. 

To unravel the contributions of various brain cells in neurodegenerative diseases, researchers have employed the brain-specific GEMs of neurons, astrocytes, and microglia, along with multi-omics data [100,101]. These models successfully replicate the metabolic interactions between these cell types in both healthy and pathological conditions. Moreover, these cell-type-specific models demonstrated the potential to reproduce observed physiological alterations, with simulations exhibiting strong agreement with experimental studies [100,101,102,103,104]. 

Adipocyte-GEM has been instrumental in studying the metabolic processes in adipocytes, which are the cells primarily responsible for storing and releasing energy as fat [28]. By integrating transcriptome and fluxome data with the adipocyte-GEM, the authors discovered specific metabolic alterations in obese subjects, including increased metabolic activity and decreased mitochondrial metabolism. Myocyte-GEM [29] was used to map transcriptional changes in T2D, uncovering significant transcriptional regulation related to pyruvate oxidation, branched-chain amino acid catabolism, and tetrahydrofolate metabolism. In another study, Zhao et al. reconstructed a comprehensive metabolic network specifically tailored to the human heart by incorporating transcriptome and proteome data [105]. By applying this heart-specific metabolic network, the authors identified novel biomarkers for cardiovascular disease (CVD) and predicted potential drug targets for different CVD subtypes. The findings from the study underscored the relevance of a heart-specific metabolic network in accurately representing the dynamic interplay between environmental factors and associated metabolic processes. 

Furthermore, by incorporating gene expression data and lipidomic experiments into HMR2, cell-specific GEMs were developed to study the activation and differentiation of CD4+ T cell subsets. The study revealed specific metabolic changes during CD4+ T cell activation and differentiation. Importantly, the significance of ceramide and glycosphingolipid biosynthesis pathways in Th17 cell differentiation was highlighted. Moreover, model predictions were validated using gene knockdown experiments, demonstrating the role of *serine palmitoyltransferase* (*SPT*) in promoting the production of proinflammatory cytokines by Th17 cells [7]. 

In addition, a comprehensive metabolic reconstruction of human small intestinal epithelial cells (sIECs) was assembled and curated [106]. The sIEC reconstructions incorporate both experimentally validated and putatively identified transporters [3,106,107]. These models have been used in studying the physiological functions of the small intestine and in gaining insights into their role in tissue metabolism. 

**Figure 1:**
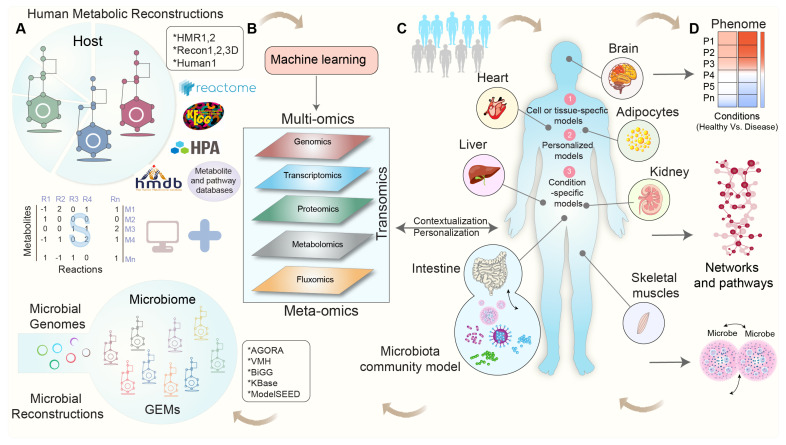
A schematic workflow for context-specific genome-scale metabolic reconstructions using multi-omics data. (**A**) Human and microbial metabolic reconstructions and pathway databases serve as a scaffold for integrating omics data, with the stoichiometric matrix (S) representing the mathematical representation of the metabolic network and capturing the stoichiometric coefficients of metabolites (M) in each reaction (R). (**B**) Various types of multi-omics and/or meta-omics datasets are employed to contextualize human and microbial metabolic reconstructions. (**C**) Cell-, tissue-, and organ-specific-GEMs are developed using metabolic reconstructions and omics datasets. (**D**) These condition-specific GEMs enable the predictions of flux phenotypes, the regulation of metabolic pathways, the identification of reporter metabolites and pathways, and the study of microbe-microbe interactions and explore the intricate relationship among diet, host, and microbiota in healthy and disease states.

In a study conducted by Gustafsson et al., they introduced a novel approach for generating cell-type models by integrating single-cell RNA sequencing (scRNA-seq) with GEMs. By clustering single-cell RNA profiles, the authors demonstrated the effectiveness of combining scRNA-seq and GEMs in advancing the understanding of human metabolism. This method shows promising potential, particularly as single-cell datasets and GEMs become increasingly accessible [108]. A schematic workflow depicting the process of generating context-specific GEMs via the integration of multi-omics data is shown in Figure 1.

Recently, multi-tissue modeling frameworks were developed to study the interactions between cell and tissue-specific models [109,110]. Bordbar et al. have developed a multi-tissue type genome-scale metabolic network for adipocytes, hepatocytes, and myocytes, allowing for the study of intercellular interactions [110]. By integrating omics data, differential metabolic activity between obese and T2D obese gastric bypass patients was examined in a whole-body context. In another study, Foguet et al. developed personalized organ-specific (multiple tissues) metabolic flux models for a large cohort; these models identified associations between the personalized flux profiles and concentrations of metabolites in the blood via a fluxome-wide association study (FWAS) [111]. Several metabolic fluxes were found to be associated with the risk of developing coronary artery disease (CAD), unravelling a mechanism. Additionally, incorporating omics data into GEM networks enables the identification of functional modules, gene regulatory networks, and metabolic interactions, contributing to a better understanding of complex metabolic pathways within cells [36,80,112]. A list of cell- and tissue-specific GEMs developed by the integration of omics datasets can be found in Table 2.

## 5. Modeling the Interactions between Gut Microbial Communities and Host Metabolism 

The emergence of high-throughput “meta”-omics technologies has significantly improved our understanding of the gut microbiome and its significance in human health and disease. Nevertheless, the vast amount of data generated by gut microbiome research demands the development of innovative computational tools and mathematical models, while approaches such as 16S rRNA amplicon sequencing and whole-genome shotgun metagenomics sequencing (WGS) have been utilized for microbiome profiling [121]; these genome-centric methods alone cannot offer mechanistic insights into individual taxa, their interactions with other gut flora, or their influence on host metabolism [13,122,123]. 

GSMM has emerged as a valuable approach for investigating the metabolic interactions between microbial communities and their host in gut ecosystems [12,15,112,124,125,126,127,128]. Recent advancements have led to the development of GEMs that are specifically tailored to catalogued human gut microbes, leveraging their metabolic functions [129,130]. One notable example is the *AGORA* (assembly of gut organisms through reconstruction and Analysis) project, which successfully conducted semi-automatic genome-scale metabolic reconstructions of 773 human gut bacteria, encompassing 205 genera and 605 species [15]. Recently, *AGORA2*, an expanded resource of human gut microbial metabolic reconstructions incorporating 7302 strains, was developed [16]. *AGORA2* facilitated personalized modeling by predicting diverse drug conversion potential in the gut microbiomes of patients with colorectal cancer vs. controls. It outperformed other resources by predicting microbial drug transformations and enabling the personalized modeling of gut microbiomes in colorectal cancer patients. By using the *AGORA* framework [15,16], metabolic interactions among microbial species were modeled by considering their metabolic capabilities and nutrient availability. *AGORA* reconstructions are publicly accessible via the *VMH* (Virtual Metabolic Human) database [60]. 

In addition, *BiGG* models [59] and the Metabolic Atlas [33,62] serve as open-access knowledge bases for genome-scale metabolic reconstructions. To facilitate the automated reconstruction of microbial GEMs, web-servers like *KBase* [97] and *ModelSEED* [61] integrate genome sequences and metagenomics datasets. Other tools, including *COMET* [131], *BacArena* [132], *dOptCom* [133], *MatNet* [134], *DyMMM* [135], *MCM* [136], and *CASINO* [126], have been developed to investigate the interplay between diets, microbiomes, and hosts. *CASINO*, for instance, successfully predicted interactions along the diet-microbiota-host axis in obesity and overweight individuals [126]. A comprehensive review discussing the implementation, characteristics, and applications of these methods can be found in [127].

Of note, the development of personalized microbiota models tailored to individual subjects is an area of recent interest. Leveraging metagenomics, meta-transcriptomics, meta-proteomics, and metabolomics data, estimating the pathway activities within the gut becomes possible, offering an approximation of the metabolic functionality specific to the gut microbes under defined conditions [137,138]. Personalized microbiota models have already demonstrated their ability to investigate secondary bile acid (BA) metabolism facilitated by human gut microbiome [128,139]. A recent study has uncovered changes in systemic BAs and microbial secondary BA pathways in infants and children who later seroconverted to multiple islet autoantibodies (i.e., being at high risk for type 1 diabetes, T1D). These findings suggest a disrupted BA metabolism that could potentially contribute to the risk and development of T1D [128]. A description of GSMM of gut microbiome including their implementation, applications, and tools are reviewed elsewhere [11,12,13,122,124,125,127,140,141].

## 6. Towards Whole-Body Metabolic Reconstruction: Bridging Precision Medicine and Systems Biology

The integration of high-throughput omics data with GEMs enables a deeper understanding of the molecular mechanisms underlying disease and facilitates the development of personalized treatment strategies. By constructing patient-specific metabolic models based on individual genomic, proteomic, and transcriptomic data, GEMs can predict metabolic alterations that are associated with specific diseases and identify potential therapeutic targets. In a study by Lewis et al. [142], personalized GEMs were developed with the aim to understand redox metabolism in cancer patients with different types of tumors. The study identified that radiation-resistant tumors produce elevated levels of reduced redox cofactors. This intriguing phenomenon suggests a potential link between redox metabolism and the ability of tumors to withstand radiation treatment. Interestingly, these models have also unveiled the presence of redox-metabolic heterogeneity among tumors that share similar clinical phenotypes. The insights provided by the personalized GEMs pave the way for more targeted approaches to cancer treatment, offering the potential for personalized interventions that consider the unique metabolic characteristics of each patient’s tumor. In another study, Turanli et al. [143] developed prostate-cancer-specific GEMs to explore cancer metabolism and identify potential therapeutic agents for effective treatment. By integrating gene expression data and a database of over 1000 drugs, the authors have predicted drug-gene interactions and identified key reactions with altered fluxes in prostate cancer. By using in silico cell viability assays, they identified potential repurposed drugs for prostate cancer treatment. Furthermore, in vitro cell assays confirmed the inhibitory effect of ifenprodil on prostate cancer cells. Agren et al. [86] employed immunohistochemistry to evaluate the presence or absence of proteins encoded by genes in 27 hepatocellular carcinoma (HCC) patients, leveraging this information, along with the *HMR 2.0* database and a task-driven model reconstruction algorithm (*tINIT*), to develop personalized GEMs for six HCC patients based on proteomics data. These personalized GEMs served as a foundation for identifying potential anticancer drugs using the concept of antimetabolites. To assess the toxicity of each antimetabolite, the authors examined the in silico functionality of 83 healthy cell-type-specific GEMs. The study predicted several antimetabolites that could effectively inhibit tumor growth in all HCC patients.

A personalized whole-body metabolic (WBM) reconstruction serves as a powerful tool that provides a comprehensive view of an individual’s metabolic profile by considering the intricate relationship between different biological systems. A WBM reconstruction enables a deeper understanding of how metabolism is influenced by factors like diet, lifestyle, and gut microbial composition; it facilitates the investigation of individual-specific responses to dietary interventions, drug treatments, and disease conditions [120]. In a groundbreaking study, Thiele et al. introduced novel WBM network reconstructions for males and females [120]. These reconstructions encompassed 26 organs, 6 blood cell types, and over 80,000 biochemical reactions, offering a comprehensive representation of whole-body organ-resolved metabolism. Notably, the WBM models successfully replicated inter-organ metabolic cycles and the patterns of energy utilization. When personalized with physiological data, they demonstrated superior predictive performance compared to existing models, particularly in estimating basal metabolic rates. Furthermore, the integration of microbiome data into these models allowed for the exploration of host-microbiome co-metabolism. 

In summary, GEMs serve as valuable tools in precision medicine, providing a multifaceted approach to understanding disease mechanisms, facilitating biomarker discovery, and guiding personalized treatment strategies that are tailored to specific patient groups.

## 7. Enhancing the Reproducibility of Genome-Scale Metabolic Models by Addressing Key Challenges

GEMs have certain limitations that need to be considered. In the context of human metabolism, GEMs face the challenge of incomplete knowledge about metabolic pathways and regulatory mechanisms. GEMs rely on the available genomic and biochemical information, which may result in gaps in the model, leading to an incomplete representation of metabolic pathways [2,26]. When it comes to microbiome modeling, a significant limitation lies in the functional characterization of the microbial species (taxa) within the gut ecosystem. Our knowledge regarding the metabolic capabilities of some individual microbes in the microbiome is incomplete, which hinders the reliability of GEMs in capturing the intricacies of microbiomes and their metabolic functions [14,127,140]. By incorporating newly available and well-annotated datasets into the existing GEMs, we can effectively address the challenges associated with the completeness of the model. Furthermore, the incorporation of multi-omics data not only facilitates the refinement and evaluation of these models but also enhances their validation, thereby increasing our confidence in the predictions. An iterative process of feedback between model predictions and experimental validation plays a crucial role in identifying and rectifying any discrepancies, enabling the continuous improvement and evolution of GEMs. This iterative process of model refinement serves as a driving force for the advancement of the models, ensuring their ability to represent complex biological systems [2]. MEMOTE (metabolic model testing) is a valuable framework designed to standardize and streamline the testing of GEMs and thereby enhance their reproducibility and credibility [144]. MEMOTE provides comprehensive test metrics to assess various aspects of model performance, including stoichiometry, thermodynamics, and network connectivity. By applying MEMOTE, researchers can compare different models and ensure the accuracy and robustness of their metabolic models. 

Another limitation is the static nature of GEMs since they primarily represent steady-state conditions and may not fully capture the dynamic and temporal changes in cellular metabolism. This means that they might not accurately model changes in metabolite concentrations and reaction rates over time [10]. To overcome this limitation, dynamic modeling approaches, such as dynamic flux balance analysis (dFBA) [145] or kinetic modeling, can be employed to capture the temporal behavior and regulatory mechanisms of metabolic networks [146,147,148]. 

Estimating parameter values in GEMs is subject to uncertainty [149]; the absence of crucial information, such as metabolite concentrations and enzyme kinetics combined with the high degrees of freedom in GEMs, presents challenges in effectively constraining these models. However, researchers have employed a range of parameter estimation and sensitivity analysis techniques to address these limitations and enhance the reliability of GEMs [149,150,151,152,153]. Notably, the *GECKO* method has been developed to construct GEMs with enzymatic constraints by incorporating kinetic and omics data [154]. This is achieved by expanding the stoichiometric matrix of the GEM to include additional information representing enzymes and their usage in metabolic reactions. Enzyme kinetics, represented by pseudo stoichiometric coefficients in the matrix, is used to model the K_cat_ values. By constraining protein abundance in this manner, *GECKO* effectively reduces flux variability and enhances the accuracy of predictions.

A common limitation of GEMs is their lack of tissue and cell-type specificity. When generic metabolic networks are reconstructed to represent specific tissues, they may not fully capture the unique metabolic characteristics of each tissue or cell-type. To address this issue and improve the contextualization of GEMs for specific conditions, the integration of multiple omics datasets becomes crucial. While algorithms have been developed to generate tissue-specific models, most rely on a single omics dataset that may not provide a comprehensive representation of cell/tissue metabolism. Therefore, there is a need for new tools and methods that effectively integrate multi-omics data to create more accurate models. Recently, a promising approach using scRNA-seq data was introduced, which provided insights into the heterogeneity and interactions within a specific cell type [108]. 

To ensure the biological relevance of GEMs, it is important to validate their predictions by comparing them against experimental data and existing knowledge. By conducting targeted experiments, researchers can assess the accuracy and reliability of the model’s predictions and gain further insights into the underlying biological processes. The experimental validation of GSMMs involves various techniques, such as in vitro assays, cell culture experiments, and animal studies. These experiments aim to measure and compare metabolic fluxes, metabolite concentrations, enzyme activities, and other relevant parameters predicted by the model against experimental observations. Using these validations, researchers can evaluate the model’s ability to capture the complex dynamics of cellular metabolism and assess its predictive power. Furthermore, the experimental validation of GSMMs also involves testing specific hypotheses generated by the model. Researchers can design experiments to manipulate specific genes, enzymes, or metabolites within the metabolic network to confirm the model’s predictions and understand the functional consequences of these perturbations.

## 8. Leveraging Machine Learning for Genome-Scale Metabolic Modeling: An Advancing Solution to Address the Key Challenges

Machine learning (ML) techniques have demonstrated their ability to identify metabolic pathways from chemical compounds; various ML methods have also been employed for metabolic pathway prediction, analysis, and modeling [155,156,157,158,159]. For instance, Li et al. developed the *mummichog* framework, which enables the direct prediction of pathway activity using spectral features from metabolomics data [160]. Another tool, *Lilikoi*, developed by AlAkwaa et al., specializes in personalized pathway-based classification modeling using metabolomics data [157]. Unlike conventional pathway tools, *Lilikoi* incorporates metabolomic profiles to generate personalized pathway profiles and identify pathways significantly associated with specific disease phenotypes. 

There is a growing interest in combining ML techniques with GSMM [8,155,156,158,161,162]. ML approaches can refine input constraints for GEMs and compare the results from simulations using experimental data [163,164]. ML methods, including linear regression (LR), decision trees, and naïve Bayes, have been used for gap-filling in draft GEMs [165,166]. Moreover, it can complement constraint-based modeling methods like FBA by incorporating additional constraints derived from omics data, such as gene expression profiles, protein abundances, and metabolite concentrations. In a recent study by Zelezniak et al. [167], the integration of ML and metabolic control analysis (MCA) was instrumental in mapping regulatory patterns and predicting cell metabolomes. The study unveiled global enzymatic changes, leading to extensive shifts in metabolic control across different sets of enzymes. 

Furthermore, GSMM and ML methods have been applied to determine metabolite secretion, flux quantification, protein turnover rate estimation, gene essentiality, metabolic gene prediction, and the assessment of a medication effect [155,156,162,163]. ML coupled with prospective GSMM has been instrumental in investigating antibiotic efficacy, sensitivity, and lethality mechanisms [168]. Guo et al. developed *DeepMetabolism*, a knowledge-based deep learning (DL) system that predicts cellular phenotypes from transcriptomics data using a combination of unsupervised and supervised approaches [169]. The framework incorporates an autoencoder with biologically guided connections and leverages FBA to evaluate the connectivity between proteomic and phenomic layers. The methodology and applications of ML for the integration of omics data into constraint-based metabolic modelling have been reviewed in detail elsewhere [159,161,170].

To summarize, ML techniques together with GSMM can unravel the complexity of biological data. ML enables the consolidation of large omics datasets, extracting key features that are linked to the desired outcomes. In contrast, GSMM offers a mechanistic comprehension, generating testable hypotheses regarding the specific metabolic processes taking place in cells, tissues, organs, or microbial communities via the incorporation of omics data. The integration of ML and GSMM holds immense promise in achieving a comprehensive understanding of human metabolism in healthy and diseased states.

## 9. Conclusions and Future Perspectives

Today, the field of GSMM has witnessed widespread application across various domains, owing to the abundance of biological data, advancements in automatic reconstruction tools, and the introduction of novel mathematical modeling techniques. As GEMs continue to evolve, they will encompass a broader range of biological pathways and gene-protein-reaction (GPR) associations. To further enhance their capabilities, incorporating additional biochemical information into GEMs becomes necessary, i.e., going beyond metabolism. This includes aspects such as protein allocation, cellular macromolecular composition, and detailed structural information about the proteins. Notably, certain molecular processes such as enzyme-substrate interactions, protein-protein complex structures, and post-translational modifications require careful consideration.

The integration of omics data into GEMs has revolutionized our understanding of biological systems. However, there are still challenges that need to be addressed to improve the reliability and reproducibility of GEM-based predictions. Efforts should be directed towards standardizing and harmonizing omics data, developing automated pipelines and tools for data integration and normalization, and incorporating ML algorithms to enhance accuracy and robustness. ML algorithms can play a crucial role in automating the reconstruction of GEMs by leveraging omics data and existing metabolic network reconstructions. Furthermore, they can be utilized to validate and refine GEMs using experimental data, ensuring an accurate representation of metabolic characteristics. ML algorithms can enable the integration of diverse omics data, allowing for the accurate prediction of active metabolic reactions under specific conditions. This is particularly important for developing high-quality models that are specific to cell types or tissues. Additionally, the incorporation of single-cell omics data enhances the accuracy of GEMs, enabling the construction of cell-type-specific models and expanding our knowledge of cellular metabolism.

In conclusion, the integration of GEMs with multi-omics data, powered by the incorporation of ML approaches, has the potential to significantly enhance our understanding of biological systems and their underlying mechanisms. These advancements pave the way for precision medicine and the development of targeted interventions for human health and disease.

## Figures and Tables

**Table 1:** Resources for microbial and human metabolic pathway reconstructions.

Databases or Repositories	Description	References
BiGG database	A publicly accessible repository for benchmark GEMs with open access.	[59]
Virtual Metabolic Human (VMH)	A freely accessible database for human and gut microbial metabolic reconstructions (GEMs) with open access.	[60]
ModelSEED	A web-based platform for metabolic modeling and analysis.	[61]
Human Metabolic Atlas (HMA)	An open access web-based platform for studying human metabolism.	[33,62]
HumanCyc	A curated database of experimentally validated metabolic pathways for studying human metabolism.	[63]
KEGG	A comprehensive resource comprising databases of large-scale molecular datasets and detailed pathway information.	[64,65]
LIPID MAPS	A database providing information on lipid structures, pathways, and lipid-related genes; enzymes; and metabolites.	[66]
Human Metabolome Database (HMDB)	A comprehensive resource that provides information on the chemical composition, biological roles, and disease associations of metabolites found in the human body.	[67]
BRENDA	An enzyme- and ligand-focused information retrieval system.	[68]
REACTOME	An open access database for biological pathways.	[69]
UniProt	An open access database for curated protein information.	[70]
Human Protein Atlas (HPA)	A comprehensive resource providing information on the expression and localization of proteins in human tissues and cells.	[71]
ProteomicsDB	A comprehensive resource for exploring and analyzing protein expression data from a variety of organisms and tissues.	[72]
Entrez gene	Gene-centered information, including gene sequences, annotations, functional data, and genetic variations.	[73]
Gene Expression Omnibus (GEO)	A public repository that provides access to a vast collection of gene expression data from various experiments and studies.	[74]
Array Express (AE)	A public database of functional genomics experiments and gene expression profiles.	[75]
European Genome-phenome Archive (EGA)	A secure and controlled-access database for hosting and sharing human genetic and phenotypic data.	[76]
Genotype-Tissue Expression (GTEx)	A catalog of genetic variants and their influence on gene expression across multiple human tissues.	[77]
Stockholm-Tartu Atherosclerosis Reverse Networks Engineering Task (STARNET)	A computational method for reconstructing cell lineage trees from single-cell transcriptomic data.	[78]
BioModels	A collection of biological models that encompasses various organisms and biological processes.	[79]

**Table 2:** Cell or tissue-specific GEMs developed using omics data. Abbreviations G, T, P, M, and F denote gene expression (microarray), transcriptomics (NGS), proteomics, metabolomics, and fluxomics, data, respectively. CVD, NAFLD, AD, T2D, T1D, MTB, IEMs, PBMCs, and HEK denote cardiovascular disease, non-alcoholic fatty liver disease, Alzheimer’s disease, type 2 diabetes, type 1 diabetes, and Mycobacterium tuberculosis, inborn errors of metabolism, peripheral blood mononuclear cells, and human embryonic kidney, respectively.

Tissue or Cell-Type	Human Metabolic Reconstructions Used for the Contextualization	Omics or Diet Data Type(s)	Phenotypes Modeled	References
Liver	HepatoNet1	G	Liver metabolism	[113]
	HMR2 (*iHepatocytes2322*)	T, P, and M	NAFLD	[6]
	HMR2	T and M	NAFLD	[8]
	Recon1	T, M, and F	NAFLD	[98]
Adipocytes	HMR1 (*iAdipocytes1809*)	T, P, and F	Adipocyte metabolism	[28]
Skeletal muscles	HMR2 (*iMyocyte2419*)	T and P	T2D	[29]
	––	T and P	CVD	[105]
Heart	Recon1 (*CardioNet*)	G	Cardiac metabolism	[114]
Brain	Astrocyte metabolic network	T	Ischemic and normal conditions	[115]
	Recon3D	T and M	AD	[103,104]
	Recon1 (*iNL403*)	G and P	AD	[101]
Kidney	––	T and P	Focal segmental glomerulosclerosis	[116]
Kidney	Recon2 (*HEK cell culture*)	––	Metabolism of HEK cells	[117]
Alveolar macrophage	Recon1 (*iAB-AMØ-1410*)	G	Host-pathogen interactions in MTB	[118]
CD4+ T-cells	HMR2	G, T, and M	CD4+ T-cells activation and differentiation	[7]
PBMCs	HMR2	G, T, and M	T1D	[119]
Human small intestinal epithelial cells	Recon1 (*hs_sIEC611*)	American diet and a balanced diet	Intestinal metabolism and IEMs	[106]
Whole-body metabolic reconstructions	Recon3D (*WBM*)	T, P, and M	Human metabolism and host-microbiome co-metabolism	[120]
